# Supplementary material for: Borehole diameter shrinkage rule considering rheological properties and its effect on gas extraction
Source: PLoS One. 2020 Sep 24;15(9):e0239016. doi: 10.1371/journal.pone.0239016 (PMC7514060; doi:10.1371/journal.pone.0239016)
Supplement: Attachment — Submitted filename: Response to Reviewers.docx [file pone.0239016.s001.docx]

Dear editor and reviewers,

Thank you very much for the valuable comments of the editor and reviewers. We have revised the manuscript and responds to each point raised by the editor and reviewers.

**Reviewer #1:**

1. Please analyze the effect of permeability on gas extraction effect.

Permeability is an important indicator that affects the difficulty of gas extraction. When a smooth extraction channel is maintained, the higher the permeability, the better the gas extraction effect; when the extraction channel is blocked, even if the coal seam permeability is high, It is also difficult to extract gas effectively.

2. It is recommended to re-consolidate the abstract of the manuscript to make it reflect the subject more accurately.

We have re-condensed the abstract of the manuscript.

3. It is recommended to modify the format of the manuscript formula to meet the style of this journal.

We revised the formula of the manuscript in accordance with the format requirements of PLOS ONE.

4. It is recommended to carefully revise the manuscript references.

We have revised the manuscript references in accordance with the format requirements of PLOS ONE.

5. Some special nouns are unified, such as "ground stress",” geo-stress”.

We have unified the nouns exclusive to the manuscript.

6. How to judge that the low gas concentration of the boreholes is caused by creep deformation but not other factors ?

Maintaining a smooth extraction channel is the key to improving gas extraction effect. The coal body around the borehole will produce creep deformation, which may block the gas extraction channel. Once the extraction channel is blocked, it is difficult to improve the extraction effect even if the extraction time is extended.

7.There are many factors that cause low gas extraction concentration, it is recommended to explain in the introduction.

The main factors affecting the concentration of gas extraction are gas extraction channels, coal seam permeability, borehole sealing quality, coal seam gas content and other factors. Various factors have been analyzed in the introduction of the manuscript.

8. There are some small errors in the paper, such as “The displacements at the junction between the the plastic softening zone and the damaged zone are equal”.It is recommended to review the paper carefully.

We carefully revised the content of the paper, found several errors, and showed them in the revised manuscript.

Reviewer #2:

In the manuscript, a visco-elastoplastic model for boreholes considering the strain softening and dilatancy and rheological properties was established, boreholes diameter shrinkage rule in soft and hard coal seams were comparatively analyzed, the reasons for the rapid reduction in the extraction concentration of the borehole in soft coal seams were described, and a technology of improving gas extraction effect of soft coal seams was developed. This topic is interesting and worth of studying, the results of manuscript are innovative. However, the manuscript needs minor revision before acceptance for publication:

1.Both permeability and borehole plugging have a large impact on the gas extraction effect. Please analyze which factor has the greater influence?

Permeability and extraction channels are the two main factors that affect the effect of gas extraction. When a smooth extraction channel is maintained, the higher the permeability, the better the gas extraction effect; when the extraction channel is blocked, even the permeability is very high and it is difficult to effectively extract gas.

2. How to determine when plugging occurs by numerical calculation?

We have established a viscoelastic-plastic mechanical model of the coal body around the borehole, obtained the expression of the borehole wall displacement, and analyzed the variation of the borehole diameter with time. When the borehole diameter is reduced to 0, the borehole is considered to be blocked.

3. There is a small mistake in section 3.3, “For a borehole with an initial diameter of 97 mm” should be “For a borehole with an initial diameter of 94 mm”.

In response to this error, we revised it in the revised manuscript.

4. There are several spelling errors, please check them carefully and improve it fluently.

We have invited Elsevier to re-edit and polish the manuscript.

Best regards.

Fuchang Hao
